# Survey for Computer-Aided Tools and Databases in Metabolomics

**DOI:** 10.3390/metabo12101002

**Published:** 2022-10-21

**Authors:** Bayan Hassan Banimfreg, Abdulrahim Shamayleh, Hussam Alshraideh

**Affiliations:** College of Engineering, Department of Industrial Engineering, American University of Sharjah, Sharjah P.O. Box 26666, United Arab Emirates

**Keywords:** metabolomics, pathway analysis, tools, computer, databases, metabolite, omics

## Abstract

Metabolomics has advanced from innovation and functional genomics tools and is currently a basis in the big data-led precision medicine era. Metabolomics is promising in the pharmaceutical field and clinical research. However, due to the complexity and high throughput data generated from such experiments, data mining and analysis are significant challenges for researchers in the field. Therefore, several efforts were made to develop a complete workflow that helps researchers analyze data. This paper introduces a review of the state-of-the-art computer-aided tools and databases in metabolomics established in recent years. The paper provides computational tools and resources based on functionality and accessibility and provides hyperlinks to web pages to download or use. This review aims to present the latest computer-aided tools, databases, and resources to the metabolomics community in one place.

## 1. Introduction

Understanding the molecular system of living organisms has led to advancements in technological techniques for measuring the function of critical biomolecules in living organisms: RNA, DNA, proteins, and small molecules of diverse natures. The analysis of such elements led to the growth of the research field known as Omics [1,2]. Omics has become the new motto of molecular biology. In recent years, the utility of Omics technologies, such as genomics, proteomics, and metabolomics [2], has delivered new perceptions of well-being.

Metabolomics enhances the monitoring of disease evolution, dietary interventions, and drug toxicities by revealing the triggers of several diseases and detecting promising links between apparently different conditions [3]. In addition, Metabolomics seeks to catch the whole set of biomolecules confined in a biological sample, creating big data explored by biostatistics and bioinformatics methods [4].

Two main challenges in Omics data analysis are the dimensionality dilemma produced by more variables than samples and the development of algorithms that successfully integrate and analyze biological data, incorporating present and future knowledge. Pathway Analysis (PA) has developed and established a reliable way of managing these issues. PA is one of the commonly used principal tools of Omics research. PA tools analyze data obtained from high-throughput technologies, identifying potentially perturbed genes in diseased samples compared to a control. In this sense, PA methods aspire to overcome the dilemma of interpreting large lists of essential genes, the main output of most basic high-throughput data analysis. In addition, PA methods provide meaning to experimental high-throughput biological data, thereby enabling interpretation and successive hypothesis generation. PA targets have been achieved by combining databases’ biological knowledge with statistical testing, mathematical analyses, and computational algorithms.

The advancement of analytical techniques and extraction methods helps detect a wide range of metabolites. The well-known analytical techniques are either mass spectrometry (MS) or nuclear magnetic resonance (NMR).

**Figure 1:**
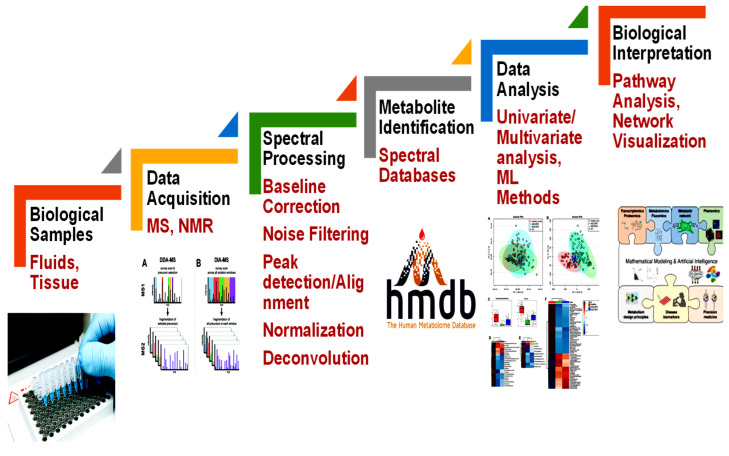
The metabolomics experiment. The flowchart comprises sample acquisitions, instrumental and spectral analysis, identifications of metabolites, and statistical and pathway analysis for further interpretation.

The conventional methodological pipeline of a metabolomics experiment combines different steps (Figure 1). This pipeline starts with biological sample acquisition to further produce metabolic information. The pre-processed metabolomics data, both MS and NMR, is typically organized into a feature quantification matrix (FQM). In this matrix, rows typically relate to the samples, while columns relate to the metabolomic features obtained. The concentration of a metabolite usually characterizes the metabolomic feature. Data analysis techniques can then be applied using these metabolomic features as input.

Medical data is mass-produced, requiring very efficient tools to manage, store, and analyze the data. Therefore, various sources are used to generate high throughput profiling of such biological and clinical data cost-effectively, such as mobile phones, sensor devices, electronic health records (EHR), patients, hospitals and clinics, researchers, and other organizations.

Big data tools in modern software systems empower remarkable research opportunities and innovation in the healthcare domain. New emerging and interrelated paradigms such as Informatics & Data-Driven Medicine [5], eHealth [6] and mHealth [7], and Digital Health [8] are booming and attaining recognition among healthcare specialists and patients.

**Figure 2:**
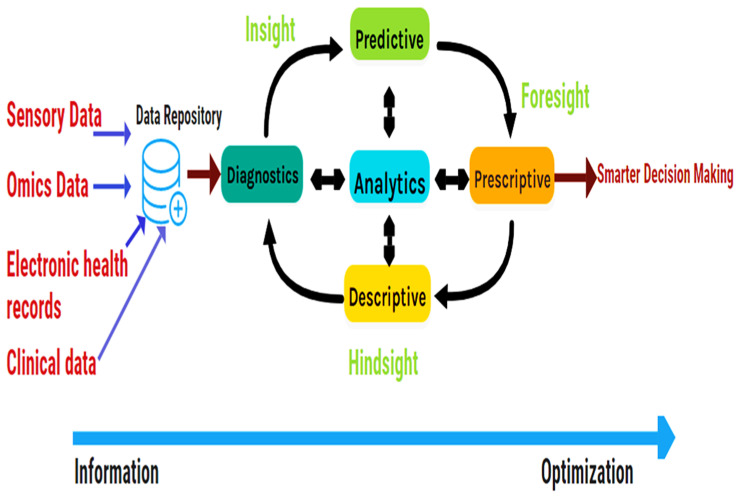
Workflow of Big data Analytics, adapted from [10]. Descriptive analytics defines what occurred. Diagnostics analytics replies why it occurred. Predictive analytics provides what will occur. Finally, prescriptive analytics proposes actions to influence desired results (make it occur).

Big Data Analytics (Figure 2) has emerged to perform descriptive and predictive analyses of such massive data. Big Data Analytics is vital and popular in bioinformatics research since the human genome size can reach 200 GB [9]. Therefore, bioinformatics researchers need to develop high-power computational algorithms and parallel programs. Thus, this study overviews primary computer-aided tools and databases in metabolomics in recent years. The report is organized into two main sections: (1) metabolomics databases and (2) computer-aided tools in metabolomics. Table 1 and Table 2 summarize all reviewed resources and their availability.

Databases store big healthcare data produced by several resources. Big data analytics platforms process the data for a better decision-making process. Descriptive analytics describes what happened. Diagnostics analytics answers why it happened. Predictive analytics gives what will happen. Finally, prescriptive analytics recommends actions to affect desirable outcomes (make it happen).

## 2. Metabolomics Databases

**Figure 3:**
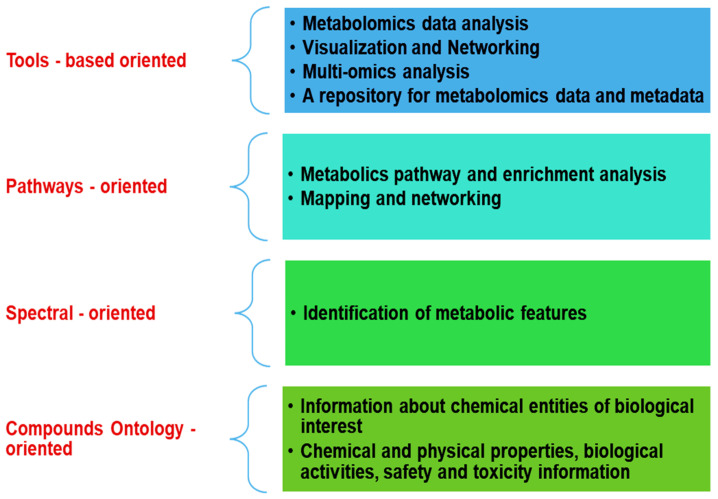
Metabolomics databases multifunctional tasks.

The vast amount of information in the ever-growing quantity of experimental and computational chemical data needs to be stored, made accessible, and manipulated. Today, hundreds of database projects are created and annotated biological knowledge. Each has a dedicated context (Figure 3).

As a result, the database’s current catalog is robust and diverse, including organism focus, curation approach, type of pathways, and interactions covered, along with other differences. In addition, many databases are available to researchers for data mining and sharing consistent chemical data for various purposes. For example, all pathway search tools depend on a database from which biochemical reactions and molecules can be enlisted to comprise the pathway of interest. This section discusses the databases related to various metabolite annotation, metabolism, and metabolomics workflows.

The Reactome Knowledgebase [11] (Reactome.org, accessed on 1 April 2022) is a distinct curated database of pathways and reactions in human biology, cross-referenced with several resources, such as essential literature and different pathway-related databases. It aims its manual annotation effort on Homo-sapiens, a single species, and applies a separate consistent data model within the whole biology domain. The Reactome describes a reaction as an event in biology that alters the condition of a biological molecule. Degradation, activation, binding, translocation, and typical biochemical events, including a catalyst, are reactions. It presents molecular features of signal transduction, transport, metabolism, DNA replication, and more cellular activities. It contains 2546 human pathways and 1940 small molecules [11].

BioCyc (Biocyc.org, accessed on 1 April 2022) [12] is a comprehensive reference to a collection of 19,494 Pathway and Genome Databases for model eukaryotes and thousands of microbes and software tools for exploring them. In addition, BioCyc comprises curated data from 130,000 publications. The MetaCyc and EcoCyc databases are freely available via BioCyc. However, access to the remaining BioCyc databases, such as HumanCyc (HumanCyc.org, accessed on 1 April 2022) [57], needs a paid subscription.

MetaCyc (MetaCyc.org, accessed on 1 April 2022) [13] is a broad metabolic pathways and enzymes database from each field of life. It includes 2937 pathways obtained from 3295 different organisms, making it the most extensive curated collection of metabolic pathways [13].

EcoCyc (EcoCyc.org, accessed on 1 April 2022) [14] is a systematic database for Escherichia coli K-12 MG1655. The EcoCyc presents a literature-based curation of its genome, transporters, metabolic pathways, and transcriptional regulation. Original and improved data analysis and visualization tools involve a circular genome viewer, an interactive metabolic network explorer, and several upgrades to the usability and speed of current tools [14]. It mainly focuses on metabolic pathways and signaling.

Metabolite Network of Depression Database (MENDA) [30] (http://menda.cqmu.edu.cn:8080/index.php, accessed on 1 April 2022) is a broad metabolite-disease association database that integrates all existing knowledge and datasets of metabolic characterization in depression. In addition, study and tissue type, organism, category of depression, sample size, platform (MS-based, MRS, NMR), and differential metabolites are provided.

BiGG Models (BIGG.ucsd.edu, accessed on 1 April 2022) [15] is a biochemical, genetic, and genomic knowledge base of genome-scale metabolic network reconstructions. BiGG Models includes more than 75 superior, manually curated genome-scale metabolic models. It also delivers a broad application interface for accessing BiGG Models with modeling and analysis kits. In addition, reaction and metabolite identifiers and pathway visualization were formalized in BiGG Models.

Kyoto Encyclopedia of Genes and Genomes (KEGG) (www.kegg.jp/, accessed on 1 April 2022) [16] is an extensive and widely used database. It is a manually curated source incorporating 18 databases classified into genomic, systems, health, and chemical data.

The Braunschweig Enzyme Database (BRENDA) enzyme database (www.brenda-enzymes.org, accessed on 1 April 2022) [17] contains comprehensive functional enzyme and metabolism data such as measured kinetic parameters. The main part has more than 5 million data points for almost 90,000 enzymes. In addition, BRENDA presents accessible enzyme information from fast to superior text- and structured-based searches for word maps, enzyme-ligand interactions, and enzyme data visualization.

PubChem (pubchem.ncbi.nlm.nih.gov, accessed on 1 April 2022) [18] is the world’s most extensive set of open and accessible chemical information from more than 750 data sources. It stores information in three primary categories: compounds, substances, and bioactivities. In addition, several research areas use PubChem as a big data resource, including machine learning and data science for drug repurposing, virtual screening, drug side effect prediction, metabolite identification, and chemical toxicity prediction. Furthermore, PubChem provides physical and chemical properties, safety and toxicity information, biological activities, literature citations, patents, and more.

ChEBI (www.ebi.ac.uk/chebi, accessed on 1 April 2022) [19] is an open-access glossary of molecular entities aimed at small biochemical compounds.

The HMDB (https://hmdb.ca, accessed on 1 April 2022) [20] is a broad source delivering information about homo-sapiens metabolites and their associated physiological, chemical, and biological properties. To date, HMDB has 220,945 total metabolites.

ChemSpider (chemspider.com, accessed on 1 April 2022) [21] is a freely accessible chemical structure database delivering a quick structure and text search covering over one hundred million structures from hundreds of data resources.

MetaboLights (https://www.ebi.ac.uk/metabolights, accessed on 1 April 2022) [22] is a database that includes metabolomics studies research, raw experimental data, and related metadata. MetaboLights is cross-technique and cross-species and includes metabolite structures and their related biological roles, reference spectra, concentrations and locations, and metabolic experiments data. Users can upload their research datasets into the MetaboLights Repository. Researchers are then automatically given a unique and stable identifier for publication reference.

The Metabolomics Workbench (metabolomicsworkbench.org, accessed on 1 April 2022) [23] is a public repository for experimental metabolomics metadata and data covering several species and experimental platforms, metabolite structures, metabolite standards, tutorials, protocols, training material, and more educational resources. It can combine, examine, deposit, track, and distribute big heterogeneous data from many MS- and NMR-based metabolomics studies. It covers over twenty diverse species, including humans and other mammals, insects, invertebrates, plants, and microorganisms.

SMPDB (https://smpdb.ca, accessed on 1 April 2022) [24] is a comprehensive, interactive, visual database that includes over 48,000 discovered pathways. Most of the pathways do not exist in other pathway databases. SMPDB help in pathway discovery and interpretation in metabolomics, proteomics, transcriptomics, and systems biology.

MetSigDis [25] (http://www.bio-annotation.cn/MetSigDis/, accessed on 1 April 2022) is a free web-based tool that offers a comprehensive metabolite alterations resource in various diseases. The database deposited 6849 curated associations between 2420 metabolites and 129 diseases among eight species, including humans and model organisms.

Virtual Metabolic Human [26] (VMH, www.vmh.life, accessed on 1 April 2022) is a web-based database capturing the knowledge of Homo-sapiens metabolism within 5 interlinked resources, including, Homo-sapiens metabolism, Disease, Gut microbiome, ReconMaps, and Nutrition. The VMH’s exceptional features are (i) the introduction of the metabolic reconstructions of Homo-sapiens and gut microbes for metabolic modeling; (ii) seven Homo-sapiens metabolic maps for data visualization; (iii) a nutrition designer; (iv) an accessible webpage and application user interface to access the content; (v) feedback option for community users’ interactions and (vi) the linking of its entities to 57 web resources.

WikiPathways [28] (wikipathways.org, accessed on 1 April 2022) is a reliable and rich pathway database that captures biological pathways’ collective knowledge. By delivering a database in a curated, machine-readable system, visualization and omics data studies is supported.

The relational database of Metabolomics Pathways (RaMP) [29] is a public database to combine biological pathways from the WikiPathways, KEGG Reactome, and the HMDB. RaMP maps metabolites and genes to biochemical and disease pathways and can be incorporated into other existing software. It can be used as a stand-alone resource (https://github.com/mathelab/RaMP-DB/, accessed on 1 April 2022) or incorporated into other tools (https://github.com/mathelab/RaMP-DB/inst/extdata/, accessed on 1 April 2022).

Pathway Commons [27] (https://www.pathwaycommons.org, accessed on 1 April 2022) is one of the most extensive composite databases. It is an integrated resource of openly accessible information about biological pathways involving biochemical reactions, transport and catalysis events, assembly of biomolecular complexes, and physical interactions, including DNA, RNA, proteins, and small molecules such as drug compounds and metabolites. A list of commonly used metabolomics databases and their main features can be found in Table 1.

A variety of databases stands as a metabolomics dataset repository. To mention some, BioMagResBank (BMRB) (http://www.bmrb.wisc.edu, accessed on 1 April 2022) [58] is a public repository for NMR spectroscopy data from peptides, proteins, nucleic acids, and more biomolecules. In addition, the Golm Metabolome Database (GMD) (http://gmd.mpimp-golm.mpg.de/, accessed on 1 April 2022) [59] provides datasets for biologically quantified active metabolites and text search capabilities for GC-MS data. Moreover, the Mass Spectral Library (https://www.NIST.gov/srd/NIST-standard-referencedatabase-1a, accessed on 1 April 2022) [60] extensively collects EI MS, MS/MS, replicate spectra, and retention index datasets. Finally, the Spectral Database System (SDBS) (https://sdbs.db.aist.go.jp/, accessed on 1 April 2022) [61] is a spectral database for organic compounds and has various MS, NMR, IR, Raman, ESR datasets.

Taken all together, Pathguide [62] is a necessary initial step for considering the prospect of pathway databases. Pathguide is a meta-database that contains information about 702 biological pathway-related databases and molecular interaction-related databases. For example, the Pathguide categories include signaling pathways, metabolic pathways, pathway diagrams, gene regulatory networks, transcription factor targets, genetic interactions networks, protein sequence-focused, protein-protein interactions, protein–compound interactions, etc.

Despite the emerging number of chemical databases, the significant challenge for this expansion is the incompetence to use metabolite and reaction information from databases such as KEGG, BRENDA, and MetaCyc because of representation inconsistencies and duplications, and errors. In addition, the same metabolite is obtained with several names among models and databases, which slows down assembling information from different data sources. Therefore, researchers designed the MetRxn database [63], Rhea [64], and RefMet [65] to standardize reaction and metabolite names. Additions and modifications to databases are made regularly to increase the quality and coverage of their biological knowledge. Some databases can update their information frequently to sustain pace with discoveries. For instance, the KEGG database [16] revises its data weekly; however, other databases do it less often. The preference of databases should consider the relative sizes, degree of overlap, and scope. For instance, KEGG comprises considerably more compounds than MetaCyc, but MetaCyc includes more pathways and reactions than KEGG. For example, pathway sets might vary between databases in several ways, involving the number of pathways present, the size of pathways, how pathways are curated, be it manually or automatically, or a combination of both, organisms supported, and the pathway boundaries [66]. However, interpreting metabolomics data has been intriguing since realizing the relationships among dozens of modified metabolites have often relied on researchers’ biochemical assumptions and knowledge. However, recent biochemical databases deliver information about metabolism’s interrelations, automatically polling using metabolomics analysis tools, i.e., mathematical and computational tools.

## 3. Metabolomics Computer-Aided Tools

**Figure 4:**
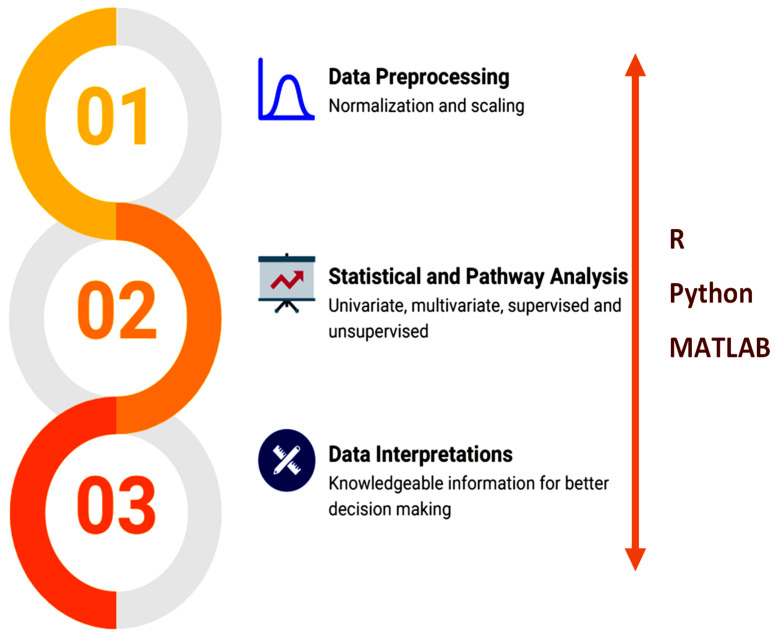
Overview of the data analysis workflow.

Python [67], R [68], and other programming languages empower and facilitate various tools to implement integrated workflows (Figure 4).

Independent computational methods for conducting statistics, enrichment, contextualization, and visualization must be combined into integrated workflows [69]. These workflows should be customized and made compatible with the study designs to attain complete and significant information from the metabolomics datasets. Mathematical methods are helpful for molecular biomarker detection. However, statistical tests, such as *t*-tests, significance analysis of microarrays (SAM), and eBayes, usually extract dysfunctional molecules from comprehensive expression data and are incorporated as an essential analytical phase in several metabolic identification pipelines. In addition, several novel computational tools have been established as secondary analysis tools to allow metabolomics researchers to grasp the powers of their data and create more beyond-achieving biological decisions than ever before. This section explains the functionality and use of various secondary analysis tools.

The MarVis-Suite [31] (http://marvis.gobics.de, accessed on 1 April 2022) (Marker Visualization) toolbox for interactive ranking, combination, filtering, visualization, clustering, and functional analysis of datasets, including intensity-based profile vectors, as found, e.g., in MS, microarray, or RNA-seq experiments.

MetExplore [32] (https://metexplore.toulouse.inra.fr/metexplore2/, accessed on 1 April 2022) offers an easy-to-use complete online solution comprised of interactive tools for metabolic network curation, network exploration, and omics data study. MetExplore holds the concepts of metabolic networks and significantly improves multi-omics data analysis.

Pathway Activity Profiling [33] (PAPi) (http://www.4shared.com/file/s0uIYWIg/PAPi_10.html, accessed on 1 April 2022) compares metabolic pathway activities from metabolite profiles. PAPi can reach the activity of metabolic pathways in several situations, which delivers excellent help for hypothesis creation and simplifies biological interpretation.

Metabolites Biological Role (MBROLE) [34] (http://csbg.cnb.csic.es/mbrole2, accessed on 1 April 2022) is a server that performs the functional enrichment research of a list of chemical compounds obtained from a metabolomics experiment, which helps the list to be explained in biological terms.

MBROLE analyzes an extensive diversity of functional annotations that define several distinct aspects of the biology and chemistry of chemical compounds; these involve pathways and sub-pathways, interactions with proteins, enzymes, and more kinds of molecules, chemical classifications and taxonomies, physiological locations, and biological functions, and applications.

MeltDB 2.0 [53] (https://meltdb.cebitec.uni-bielefeld.de, accessed on 1 April 2022) is a next-generation web application delivering storage, standardization, sharing, integration, and the analysis of metabolomics assays.

MetaboAnalyst version 5.0 [35] (https://www.metaboanalyst.ca, accessed on 1 April 2022) is a fully automated web interface to bridge raw data to functional insights for global metabolomics based on high-resolution mass spectrometry (HRMS). MetaboAnalyst performs enhanced peak detection, annotation tasks, and alignment for LC-MS data produced in global metabolomics. The key features of MetaboAnalyst are that it includes: (1) the MetaboAnalystR package in the R environment, (2) large libraries for metabolite sets and metabolic pathways, (3) metabolomic biomarker metanalysis, (4) the integration of multi-omics data over visualization and knowledge-based network analysis, and (5) an easy and free, accessible tool.

Metabolite pathway enrichment analysis (MPEA) [36] (http://ekhidna.biocenter.helsinki.fi/poxo/mpea/, accessed on 1 April 2022) is a metabolomics pathway enrichment tool for visualization and biological interpretation. MPEA is limited to top-down/bottom-up analysis. MetaP-server [55] (http://metabolomics.helmholtz-muenchen.de/metap2/, accessed on 1 April 2022) is a user-friendly web-server-based for metabolomics data analysis. It covers data acquisition to biological interpretation: (i) data quality checks, (ii) estimate of reproducibility and batch effects, (iii) hypothesis assessments for several categorical phenotypes, (iv) correlation analyses for metric phenotypes, (v) optionally involving all potential sets of metabolite concentration ratios, (vi)mapping of metabolites against colored KEGG pathway maps and (vii) PCA.

Mass TRanslator into Pathways (MassTRIX) [54] (www.masstrix.org, accessed on 1 April 2022) annotates metabolites in high-precision MS data. It marks the discovered chemical compounds on KEGG pathway maps using the KEGG/API. In addition, genes or enzymes can be underlined to denote information on gene transcription or differences in the gene complement of several bacterial strains.

Pathos [56] (http://motif.gla.ac.uk/Pathos/, accessed on 1 April 2022) is a web-based tool for analyzing raw or processed metabolomics mass spectra and demonstrating the metabolites identified and alterations in their experimental abundance within the context of their associated metabolic pathways. Pathos is limited to specific organism databases.

PaintOmics 3 [37] (www.paintomics.org, accessed on 1 April 2022) is a web-based tool for the integrated visualization of several omic data types onto KEGG pathway diagrams. PaintOmics 3 combines server-end abilities for data analysis with the capability of modern web resources for data visualization, delivering investigators with a robust framework for an interactive examination of their multi-omics information.

IMPaLA [38] (http://impala.molgen.mpg.de, accessed on 1 April 2022) is a web-based tool for joint pathway analysis with expression (genes/proteins) and metabolite data. It performs enrichment analysis or over-representation with user-specified lists of genes and metabolites utilizing more than three thousand pre-annotated pathways from eleven databases.

MetaMapR [39] (http://dgrapov.github.io/MetaMapR/, accessed on 1 April 2022) is a free-source, web-based, or desktop software employed in the R programming language. It incorporates enzymatic transformations with metabolite structural similarity, mass spectral similarity, and empirical relationships to create well-associated metabolic networks.

The Layered Enrichment Analysis of Pathways (LeapR) [40] (https://github.com/biodataganache/leapR, accessed on 1 April 2022) is a framework to measure biological pathway activity utilizing various statistical analyses and data resources, permitting facile incorporation of multisource data.

PAthway NEtwork Visualizer (PANEV) [41] (https://github.com/vpalombo/PANEV, accessed on 1 April 2022) is an R package for gene or pathway-based network visualization. Using KEGG, it visualizes genes within a network of multiple levels of interlinked upstream and downstream pathways. The network graph visualization facilitates interpreting the functional profiles of gene clusters. However, PANEV is a KEGG-based tool that can be considered a limitation because of KEGG’s lack of or incomplete information.

PathfindR [42] is an R package using protein-protein interaction information and active-subnetwork-oriented pathway enrichment analyses for class comparison omics experiments. It also provides functionality for clustering the resulting pathways.

Ingenuity Pathway Analysis [43] (IPA, http://www.ingenuity.com, accessed on 1 April 2022) is a comprehensive visualization software or database search tool for discovering functions and pathways for specific biological conditions. IPA helps realize complex omics data and achieve insightful data analysis and interpretation by putting experimental results in the context of biological systems. Its pathway focuses on protein-protein interactions, protein–compound interactions, metabolic, signaling, gene regulation, and diagrams.

iPath3.0 [44] (http://pathways.embl.de, accessed on 1 April 2022) is a free web-based tool for visualization, customization, and analysis of various KEGG cellular pathways. In addition, version 3 can deal with metabolic and regulatory pathways and the biosynthesis of secondary metabolites.

ReactomePA [45] (http://www.bioconductor.org/packages/ReactomePA, accessed on 1 April 2022) is a free R/Bioconductor package delivering enrichment analyses involving gene set enrichment analyses and hypergeometric tests. For example, functional analysis can be applied to the genomic coordination taken from a sequencing experiment to explore a genomic loci’s functional significance, including non-coding regions and cis-regulatory elements. In addition, ReactomePA offers various visualization functions to generate very customizable, publication-quality figures.

MetExploreViz [46] (http://metexplore.toulouse.inra.fr/metexploreViz/doc/, accessed on 1 April 2022) is an open-source web component for visualizing pathways and metabolic networks and presents a solution to examine omics data in a biochemical perspective.

Recon3D [47] (http://vmh.life, accessed on 1 April 2022and http://bigg.ucsd.edu/, accessed on 1 April 2022) is a computational source that comprises protein structure data and three-dimensional (3D) metabolite and allows integrated analyses of metabolic functions in humans. Recon3D is the most comprehensive human metabolic network model, reporting 3288 open reading frames (representing 17% of functionally annotated human genes), including 12,890 protein structures, 4140 unique metabolites, and 13,543 metabolic reactions. These data offer an outstanding resource for examining molecular mechanisms of human metabolism.

ChemRICH [48] (www.chemrich.fiehnlab.ucdavis.edu, accessed on 1 April 2022 and www.github.com/barupal/chemrich, accessed on 1 April 2022) is a statistical enrichment method relying on chemical similarity instead of sparse biochemical knowledge annotations. ChemRICH utilizes chemical ontologies and structure similarity to map all known metabolites and name metabolic modules. Unlike pathway mapping, this strategy generates research-specific, non-intersecting groups of all identified metabolites.

KEGGREST [49] (bioconductor.org/packages/release/bioc/html/KEGGREST.html, accessed on 1 April 2022) is an R package employed to build an adjacency matrix that links the dataset’s metabolites with their matching KEGG pathways. First, one is allocated if the metabolite is part of that specific pathway or 0 if not. The following five metabolites of each pathway were at random samples.

MetaX [50] offers several functions: peak picking and annotation, data quality assessment, missing and zero values imputation, data standardization/normalization, univariate and multivariate statistical analysis, power analysis and sample size estimate, receiver operating characteristic (ROC) analysis, biomarkers selection, and pathway annotation, correlation network analysis, and metabolite identification. It is available as a web-based interface and R package (http://metax.genomics.cn, accessed on 1 April 2022).

Biomarker Discovery by Machine Learning (BioDiscML) [51] (https://github.com/mickaelleclercq/BioDiscML, accessed on 1 April 2022) is a biomarker discovery tool that exploits several features for selection methods to generate signatures coupled with machine learning models that will predict a particular outcome efficiently. BioDiscML employs a massive selection of machine-learning algorithms to choose the ultimate combination of biomarkers for expecting continuous and categorical results from very unbalanced datasets. BioDiscML can implement data pre-processing, features and model selection, and performance assessment. The software tool is developed in JAVA 8 language and uses the Weka 3.8 machine learning library. It outperforms recent tools for discovering biomarkers’ signatures.

ASICS [70] is an R package that covers a full workflow to analyze spectra from NMR experiments. It includes an automatic method to identifying and quantifying metabolites in a complex mixture spectrum and utilizes the quantification outcomes in untargeted and targeted statistical experiments. However, ASICS has algorithm limitations: the difficulty in detecting the low concentration metabolites or their peaks, all placed in an area with a high density of peaks.

3Omics [52] (http://3omics.cmdm.tw, accessed on 1 April 2022) is a web-based visualization tool incorporating human metabolomic, transcriptomic, and proteomic data. It produces inter-Omics correlation networks to visualize data associations for all metabolites, transcripts, and proteins for time or experimental situations.

Also, one study [71] examined about 100 metabolomics software sources, databases, tools, and more utilities that have emerged or been enhanced in 2019. Similarly, around 85 metabolomics software sources, tools, packages, databases, and other utilities that appeared in 2020 were released in a recent study [72]. Finally, Table 2 surveyed commonly used metabolomics tools in the literature.

Each tool has strengths and weaknesses and should not come from using one over the other. Due to the complexity of metabolomics data, it is essential to regard the outcomes from the secondary analysis with caution. For example, enrichment analysis can generate significant pathway hits from only one or two metabolites in a pathway. As such, precise scrutinization and logical biological interpretation of the data should be undertaken. With this in mind, metabolomics scientists should incorporate secondary analysis into their analyses, as these beneficial outcomes can be attained rapidly [73]. The secondary analysis field is coming into its own, and its steady growth will help enhance the success of the metabolomics approach. These cutting-edge bioinformatics analysis tools that are completely incorporated with various functions and are accessible and manageable by users who lack prior knowledge in programming are vital in metabolomics research. They will persist in enabling discoveries and more significant insights for increasing metabolomics research.

## 4. Discussion and Concluding Remarks

This paper has highlighted extensive lists of metabolomics databases and computer-aided tools.

Databases are considered the cornerstone in metabolomics assays, and choosing a database could substantially affect the results. During recent decades, advancements in metabolomics databases have caused several formalization schemes, impeding the interoperability among these resources and generating data silos. In addition, metabolomics database selection, metabolite misidentification rate, and assay chemical bias of several analytical platforms will impact subsequent methods. Therefore, the suggestion to overcome the database’s pitfalls is to perform organism-specific metabolomics analysis using multiple databases and form a consensus signature using the outcomes. Databases integration comprising multiple databases, such as the ConsensusPathDB [74] or PathMe [75], might be helpful and consider continuing attempts to standardize the different resources.

A critical overview of the performance of selected bioinformatics tools for omics datasets is presented for the first time. These tools include BioCyc/HumanCyc [12], ConsensusPathDB [72], MBRole [34], IMPaLA [38], Metabox [76], MetaboAnalyst [35], MetExplore [32], MPEA [36], Reactome [11], PathVisio [77], and KEGGREST [49]. Despite the tool’s variability, they generated coherent outcomes independent of their analytical method. Nevertheless, further effort on the completeness of metabolomics databases is necessary, dramatically impacting the accuracy of the analysis.

Computer-aided tools are evolving, and recently, an abundance exists for metabolomics researchers [78]. Moreover, these tools have promising features to elevate metabolomics research [79,80]. User-friendly, open access and instant results are desirable attributes.

However, high-quality data analysis tools are crucial for repeatability, reproducibility, and minimal uncertainty. An experiment should generate similar responses using the same inputs; otherwise, there is little promise that an algorithm can be predictive. Therefore, the available tools should be classified based on performance; however, lacking a measure to validate their performance. Few studies have attempted to compare and measure various tools’ performance, yet more efforts are required to embrace these tools with certainty.

Metabolomic technological capabilities and data sharing, for instance, database incorporation, will be crucial in the future expansion of metabolomics and enable enhancements in multi-organism systems biology.

## Figures and Tables

**Table 1:** Summary of metabolomics databases.

Database	Organisms	Database Descriptions	Coverage	Accessibility	Link	Y.O.R	Ref.
Reactome Knowledgebase	Homo-sapiens	It contains visualization, interpretation, and analysis of pathway knowledge. Available tools: SkyPainter, PathFinder, BioMart, Reactome Gene Set Analysis (ReactomeGSA) and Reactome IDG Portal.	Human Pathways:2546 Reactions:13890 Proteins:1020 Small Molecules:1940 Drugs:507	Free	Reactome.org (accessed on 1 April 2022)	2005	[11]
BioCyc	Eukaryotes Bacteria and Archaea.	A comprehensive reference containing listed data from 130,000 publications—available tools: Pathologic, Genome browser, Pathway Tools, BLAST search, and SmartTables.	Pathway/Genome Databases (PGDBs): 19,494 Archaea: 465 databases Bacteria: 18,956 databases Eukaryota: 37 databases MetaCyc: Metabolic Encyclopedia	EcoCyc and MetaCyc databases: free access. Others: Paid subscription	Biocyc.org (accessed on 1 April 2022)	1997	[12]
MetaCyc	Eukaryotes Bacteria and Archaea.	Serves as a comprehensive reference to metabolic pathways and enzymes. Available tools: Pathologic, Genome browser, BLAST search, Pathways Tools, Google™.	Multi-organisms: 3295 Metabolic pathways:2937 Enzymatic reactions:17,310	Free	MetaCyc.org (accessed on 1 April 2022)	1999	[13]
EcoCyc	Bacterial organism: Escherichia coli K-12 MG1655	Contains Metabolic Network Explorer, Circular Genome Viewer	Genes:4518 Enzymes:1682 Metabolic reactions:2151	Free	EcoCyc.org (accessed on 1 April 2022)	1995	[14]
BIGG Models	Eukaryotes, Prokaryotes, and Photosynthetic Eukaryotes.	Provides pathway visualization with Escher. It also offers standardized identifiers for metabolites, reactions, and genes.	It contains more than 75 high-quality manually-curated genome-scale metabolic models.	Free	BIGG.ucsd.edu (accessed on 1 April 2022)	2007	[15]
KEGG	Eukaryotes Bacteria and Archaea.	PATHWAY database, KEGG NETWORK database, KO annotation and taxonomy, drug information, and virus-cell interaction. Available tools: KEGG Atlas, KegHier, KegArray, KegDraw, KegTools, KEGG2, KEGG API.	KEGG organisms: 7760 (Eukaryotes: 695, Bacteria:6694, Archaea:371). KEGG modules: 456 Reaction modules:46	Free	www.kegg.jp/ (accessed on 1 April 2022)	1995	[16]
BRENDA	Eukaryotes Bacteria and Archaea.	Comprises disease-related data, protein sequences, 3D structures, genome annotations, ligand information, taxonomic, bibliographic, and kinetic data.	Number of different enzymes: 8197	Free	www.brenda-enzymes.org (accessed on 1 April 2022)	1987	[17]
PubChem	Eukaryotes Bacteria and Archaea	Provides chemical and physical properties, biological activities, safety and toxicity information, patents, literature citations, and more. Available tools: PubChem Structure Editor, Entrez, PubChem3D, PubChem Download Facility, ToxNet.	Compounds:110 million, Substances:277 million, Bioactivities:293 million.	Free	PubChem.ncbi.nlm.nih.gov (accessed on 1 April 2022)	2004	[18]
ChEBI	Eukaryotes Bacteria and Archaea	A database and ontology containing information about chemical entities of biological interest.	Annotated compounds: 59,708	Free	www.ebi.ac.uk/chebi (accessed on 1 April 2022)	2010	[19]
HMDB	Homo-sapiens	A human metabolomics database. It has spectral and pathway visualization tools. Available tools: Data Extractor, ChemSketch, BLAST search, MetaboCard, MS and NMR spectral search utility, MetaboLIMS.	Annotated metabolite entries: 217,920	Free	https://hmdb.ca (accessed on 1 April 2022)	2007	[20]
ChemSpider	Eukaryotes Bacteria and Archaea	A chemical structure database.	Chemical entities:114 Million	Free	chemspider.com (accessed on 1 April 2022)	2007	[21]
MetaboLights	Eukaryotes Bacteria and Archaea	An open-access database repository for cross-platform and cross-species metabolomics research.	Different organisms: 6510 Reference compounds:27,475 Metabolite annotation features:2016,457	Free	https://www.ebi.ac.uk/metabolights (accessed on 1 April 2022)	2012	[22]
Metabolomics Workbench	Eukaryotes Bacteria and Archaea	A repository for metabolomics data and metadata and provides analysis tools and access to metabolite standards, protocols, tutorials, training, and more.	Discrete structures:136,000 Genes:7300 Proteins:15,500	Free	metabolomicsworkbench.org (accessed on 1 April 2022)	2016	[23]
SMPDB	Eukaryotes Bacteria and Archaea	A pathway database for different model organisms such as humans, mice, E. coli, yeast, and Arabidopsis thaliana.	Pathways Number: 48,690 Metabolites Number (non-redundant): 55,700	Free	https://smpdb.ca/ (accessed on 1 April 2022)	2009	[24]
MetSigDis	Homo-sapiens, Rat, Mouse, Drosophila melanogaster, Triatomine, Mice, Pig, and Mus musculus.	A manually curated resource that aims to provide a comprehensive resource of metabolite alterations in various disease.	Curated relationships:6849 Metabolites:2420 Diseases:129 Species: 8	Free	http://www.bio-annotation.cn/MetSigDis/ (accessed on 1 April 2022)	2017	[25]
Virtual Metabolic Human	Homo-sapiens	Captures human and gut microbial metabolism information and links it to hundreds of diseases and nutritional data.	Reactions:19,313 Metabolites:5607 Human genes:3695 Diseases:255 Foodstuff:8790	Free	www.vmh.life (accessed on 1 April 2022)	2018	[26]
Pathway Commons	Eukaryotes Bacteria and Archaea	Aims to collect and disseminate biological pathway and interaction data	Pathways:5772 Interactions:2,424,055 Databases:22	Free	https://www.pathwaycommons.org (accessed on 1 April 2022)		[27]
WikiPathways	Eukaryotes Bacteria and Archaea	A public, collaborative platform devoted to the curation of biological pathways	Human genes: 11,532 Number of pathways: 3013	Free	wikipathways.org (accessed on 1 April 2022)	2008	[28]
RaMP	Eukaryotes Bacteria and Archaea	A multi-database integration approach for gene/metabolite enrichment analysis providing interactive tables of query results, interactive tables of pathway analysis results, and clustering of enriched pathways by pathway similarity	Pathways: 51,526 (from KEGG, Reactome, SMPDB, and WikiPathways) Genes: 23,077 Metabolites: 113,725	Free	https://github.com/mathelab/RaMP-DB/orhttps://github.com/mathelab/RaMP-DB/inst/extdata/ (accessed on 1 April 2022)		[29]
MENDA	Organisms include: Human, Rat, Mouse, and Non-human primates.	A comprehensive metabolic characterization database for depression.	Differential expressed metabolites: 5675. (Humans:1347 Rat:3127 Mouse:1105 Non-human primates:96)	Free	Menda.cqmu.edu.cn:8080/index.php\ (accessed on 1 April 2022)	2020	[30]

**Table 2:** Summary of computer-aided metabolomics.

Tool Name	Description	Input	Implementation	Accessibility	Databases Used	Link	Ref
MarVis-Suite	Metabolic pathways analysis and visualization	MS, microarray, or RNA-seq experiments	Web-based	Free	KEGG and BioCyc	http://marvis.gobics.de (accessed on 1 April 2022)	[31]
MetExplore	Metabolic network and OMICs data analysis	Any	Web-based	Free	BioCyc- related	https://metexplore.toulouse.inra.fr/metexplore2/ (accessed on 1 April 2022)	[32]
PAPi	Compare activity of metabolic pathway between sample types.	Any	R package	Free	KEGG	http://www.4shared.com/file/s0uIYWIg/PAPi_10.html (accessed on 1 April 2022)	[33]
MBROLE	Enrichment analysis of metabolites annotations.	Any	Web-based	Free	KEGG, HMDB, PubChem, ChEBI, SMILES, YMDB, ECMDB, BioCyc-related, Rhea, UniPathway, LMSD, CTD, MeSH, MATADOR, DrugBank.	http://csbg.cnb.csic.es/mbrole2 (accessed on 1 April 2022)	[34]
MetaboAnalyst 5.0	Metabolomics analysis platform, tutorials, and report analysis.	LC, GC raw spectra, MS, NMR peak list, and spectral bins.	Web-based, R package	Free	KEGG, HMDB, PubChem, ChEBI, RefMet and LIPID MAPS.	https://www.metaboanalyst.ca (accessed on 1 April 2022)	[35]
MPEA	Pathway enrichment analysis.	Pre-annotated compounds or GC-MS-based MSTs	Web-based	Free	KEGG, SMPDB and GMD.	http://ekhidna.biocenter.helsinki.fi/poxo/mpea/ (accessed on 1 April 2022)	[36]
PaintOmics 3	Compound mapping	Any	Web-based	Free	KEGG	www.paintomics.org (accessed on 1 April 2022)	[37]
IMPaLA	Enrichment analysis.	Any	Web-based	Free	Reactome, KEGG, Wikipathways, HMDB, CAS, ChEBI, PubChem, SMPDB, NetPath, BIOCART, BioCyc.	http://impala.molgen.mpg.de (accessed on 1 April 2022)	[38]
MetaMapR	Metabolic network mapping.	LC and GC raw spectra, MS and NMR peak list, and spectral bins.	Web-based or desktop software.	Free	KEGG and PubChem	http://dgrapov.github.io/MetaMapR/ (accessed on 1 April 2022)	[39]
LeapR	Enrichment analysis.	Any	R package	Free		https://github.com/biodataganache/leapR (accessed on 1 April 2022)	[40]
PANEV	Gene/pathway-based network visualization	Any	R package	Free	KEGG	https://github.com/vpalombo/PANEV (accessed on 1 April 2022)	[41]
PathfindR	Enrichment analysis.	Any	R package	Free	KEGG, Biogrid, v, IntAct,	https://cran.r-project.org/package=pathfindR (accessed on 1 April 2022)	[42]
Ingenuity Pathway Analysis	Metabolic network mapping.	Any	Web-based, software	Paid	GO, KEGG, BIND	IPA, http://www.ingenuity.com (accessed on 1 April 2022)	[43]
iPath3.0	Metabolic network mapping.	Compound IDs	Web-based	Free	KEGG, Uniprot, STRING, protein IDs, COGs, eggNOGs, NCBI gene identifiers, ChEBI and PubChem.	http://pathways.embl.de (accessed on 1 April 2022)	[44]
ReactomePA	Enrichment analysis.	Any	R-package	Free	REACTOME	http://www.bioconductor.org/packages/ReactomePA (accessed on 1 April 2022)	[45]
MetExploreViz	Metabolic network mapping.	Any	Web-based	Free	KEGG	http://metexplore.toulouse.inra.fr/metexploreViz/doc/ (accessed on 1 April 2022)	[46]
Recon3D	Network reconstruction	Any	Web-based	Free	KEGG, PDB, CHEBI, PharmGKB, UniProt	http://vmh.life (accessed on 1 April 2022)	[47]
ChemRICH			Web-based and R-package	Free	NCBI BioSystems, PubChem, KEGG, BioCyc, Reactome, GO, and Wikipathways	www.chemrich.fiehnlab.ucdavis.edu) and www.github.Com/barupal/chemrich (accessed on 1 April 2022)	[48]
KEGGREST	A package providing a client interface to the KEGG REST server.	Compound IDs	R package	Free	KEGG	https://bioconductor.org/packages/release/bioc/html/KEGGREST.html (accessed on 1 April 2022)	[49]
MetaX	Flexible and comprehensive Software for processing metabolomics data	Raw peak intensity data	Web-based and R-package	Free	HMDB, KEGG, MassBank, Pub-Chem, LIPID MAPS, MetaCyc, and PlantCyc	http://metax.genomics.cn). (accessed on 1 April 2022)	[50]
BioDiscML	Biomarker discovery software that supports classification and regression problems.	Any	Stand-alone program	Free		https://github.com/mickaelleclercq/BioDiscML. (accessed on 1 April 2022)	[51]
3Omics	Web tool visualization of multi-omics data (transcriptomics, proteomics, and metabolomics)	Any	Web-based	Free	iHOP, KEGG, HumanCyc, DAVID, Entrez Gene, OMIM and UniProt	http://3omics.cmdm.tw (accessed on 1 April 2022)	[52]
MeltDB 2.0	Web-based tool for statistical analysis and sets for enrichment analysis.	Raw GC/LC-MS spectra, processed spectra, compound IDs, and abundances.	Web-based, login required	Free	KEGG, ChEBI, GMD and CAS.	https://meltdb.cebitec.uni-bielefeld.de (accessed on 1 April 2022)	[53]
MassTRIX	Compound mapping	MS spectra	Web-based	Free	KEGG, HMDB and LipidMaps.	www.masstrix.org (accessed on 1 April 2022)	[54]
MetaP-server	Global statistical analysis	Compound IDs and sample metadata.	Web-based	Free	KEGG, HMDB, LIPID MAPS, PubChem and CAS.	http://metabolomics.helmholtz-muenchen.de/metap2/) (accessed on 1 April 2022)	[55]
Pathos	Compound mapping	MS-spectra (raw *m/z*) and compound IDs (KEGG or MetaCyc IDs)	Web-based	Free	KEGG	http://motif.gla.ac.uk/Pathos/) (accessed on 1 April 2022)	[56]
